# Supplementary material for: Isoindigo–Thiophene D–A–D–Type Conjugated Polymers: Electrosynthesis and Electrochromic Performances
Source: Int J Mol Sci. 2023 Jan 22;24(3):2219. doi: 10.3390/ijms24032219 (PMC9916795; doi:10.3390/ijms24032219)
Supplement: Supplementary file 1 [file ijms-24-02219-s001.zip › ijms-2144736-supplementary.pdf]

Supplementary Information

Isoindigo-thiophene D-A-D type conjugated polymers: electrosynthesis and electrochromic performances

Jie Cao[1,2]†, Xiaoyu Luo[1,3]†, Shenglong Zhou[1,2], Zhixin Wu[1,2], Qi Zhao[1,3], Hua Gu[1,2], Wen Wang[1,2], Zhilin Zhang[1,3], Kaiyue Zhang[1,3], Kaiyun Li[1,2], Jingkun Xu[1,2,4], Ximei Liu[1,2], Baoyang Lu[1,3]*, Kaiwen Lin[5]*

[1] *Jiangxi Key Laboratory of Flexible Electronics, Flexible Electronics Innovation Institute, Jiangxi Science and Technology Normal University, Nanchang 330013, China*

[2] *School of Chemistry and Chemical Engineering, Jiangxi Science and Technology Normal University, Nanchang 330013, China*

[3] *School of Pharmacy, Jiangxi Science and Technology Normal University, Nanchang 330013, Jiangxi, PR China*

[4] *School of Chemistry and Molecular Engineering, Qingdao University of Science and Technology, Qingdao 266042, China*

[5] *Department of Materials and Food, University of Electronic Science and Technology of China Zhongshan Institute, Zhongshan 528402, China*

* Corresponding authors. Tel: +86-791-88537967; Fax: +86-791-83823320.

Email: luby1258@163.com; kevinlin1990@163.com

†: These authors contributed equally to this work.

Contents

Figure S1. ^{1}H NMR spectrum of (*E*)-6,6'-dibromo-[3,3'-biindolinylidene]-2,2'-dione in $CDCl_3$.

Figure S2. ^{1}H NMR spectrum of (*E*)-6,6'-dibromo-1,1'-dihexyl-[3,3'-biindolinylidene]-2,2'-dione in $CDCl_3$.

Figure S3. ^{1}H NMR spectrum of (*E*)-6,6'-dibromo-1,1'-dihexyl-[3,3'-biindolinylidene]-2,2'-dione in $CDCl_3$.

Figure S4. ^{1}H NMR spectrum of IDOH-Th in $CDCl_3$.

Figure S5. ^{1}H NMR spectrum of IDOD-Th in $CDCl_3$.

Figure S6. ^{13}C NMR spectrum of IDOD-Th in $CDCl_3$.

Figure S7. ^{1}H NMR spectrum of IDOH-3HT in $CDCl_3$.

Figure S8. ^{13}C NMR spectrum of IDOH-3HT in $CDCl_3$.

Figure S9. ^{1}H NMR spectrum of IDOD-3HT in $CDCl_3$.

Figure S10. Chemical structures of different D-A-D type precursors.

Figure S11. FT-IR spectra of precursors IDOH-Th (A), IDOD-Th (B), IDOH-3HT (C), IDOD-3HT (D), and corresponding polymers.

Figure S12. SEM images of PIDOH-Th and PIDOD-Th were deposited electrochemically on the ITO electrode: dedoped PIDOH-Th (A) and doped PIDOH-Th (B); dedoped PIDOD-Th (C) and doped PIDOD-Th (D).

Figure S13. SEM images of PIDOH-3HT and PIDOD-3HT deposited electrochemically on the ITO electrode: dedoped PIDOH-3HT (A) and doped PIDOH-3HT (B); dedoped PIDOD-3HT (C) and doped PIDOD-3HT (D).

Figure S14. Long-term CVs of the deposited PIDOH-3HT film at a constant potential scan rate of 200 mV s^{-1}.

Table S1. Peak assignments and comparison between observed peaks of ITPs and corresponding polymers.

(*E*)-6,6'-dibromo-[3,3'-biindolinylidene]-2,2'-dione: 92%. ^{1}H NMR (400 MHz, $CDCl_3$, ppm) δ 11.09 (s, 2H), 8.97 (d, J = 8.6 Hz, 2H), 7.17 (d, J = 8.6 Hz, 2H), 6.99 (s, 2H).

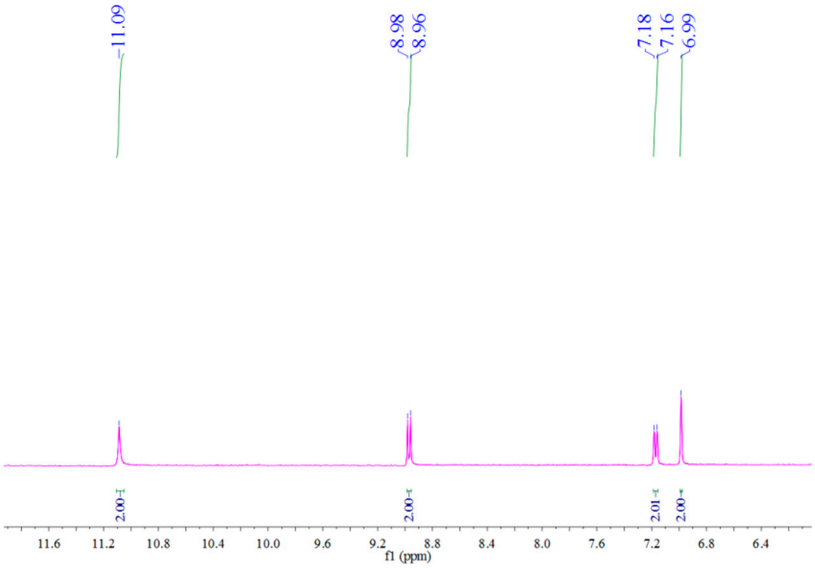

Figure S1. ^{1}H NMR spectrum of (*E*)-6,6'-dibromo-[3,3'-biindolinylidene]-2,2'-dione in $CDCl_3$.

(*E*)-6,6'-dibromo-1,1'-dihexyl-[3,3'-biindolinylidene]-2,2'-dione: 81%. ^{1}H NMR (400 MHz, $CDCl_3$, ppm) δ 9.09-9.07 (d, *J* = 8.6 Hz, 2H), 7.19-7.17 (dd, *J* = 8.6 Hz, 2H), 6.94 (s, 2H), 3.76-3.74 (t, *J* = 7.4 Hz, 4H), 1.73-1.65 (m, 4H), 1.36-1.26 (s, 12H), 0.90-0.88 (t, *J* = 6.8 Hz, 6H).

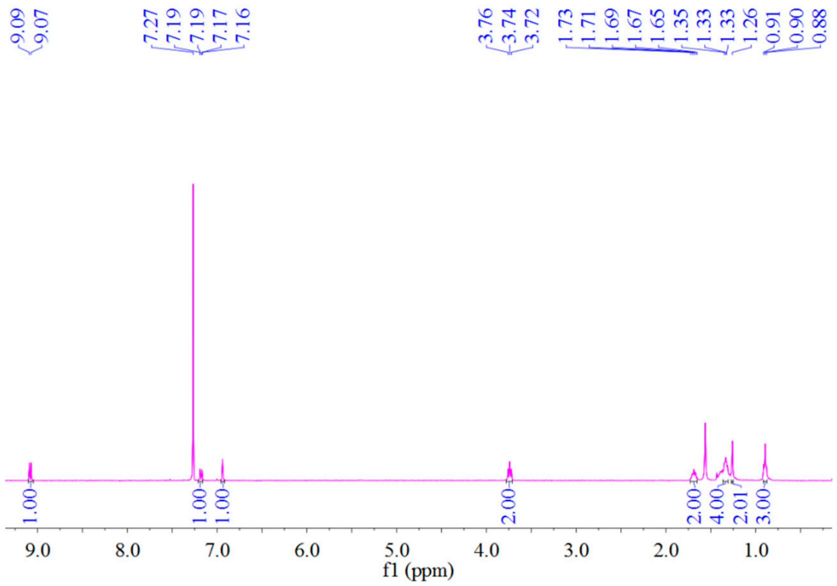

Figure S2. ^{1}H NMR spectrum of (*E*)-6,6'-dibromo-1,1'-dihexyl-[3,3'-biindolinylidene]-2,2'-dione in $CDCl_3$.

(*E*)-6,6'-dibromo-1,1'-dihexyl-[3,3'-biindolinylidene]-2,2'-dione: 56%. ^{1}H NMR (400 MHz, $CDCl_3$, ppm) δ 9.20-9.17 (d, *J* = 8.6 Hz, 2H), 7.45 (dd, *J* = 8.6 Hz, 2H), 7.37 (s, 2H), 7.34-7.13 (t, *J* = 6.4 Hz, 2H), 6.69 (s, 2H), 3.85-3.81 (t, *J* = 9.4 Hz, 4H), 1.76-1.72 (m,4H), 1.36-1.26 (s, 58H), 0.88-0.85 (t, *J* = 6.8 Hz, 10H).

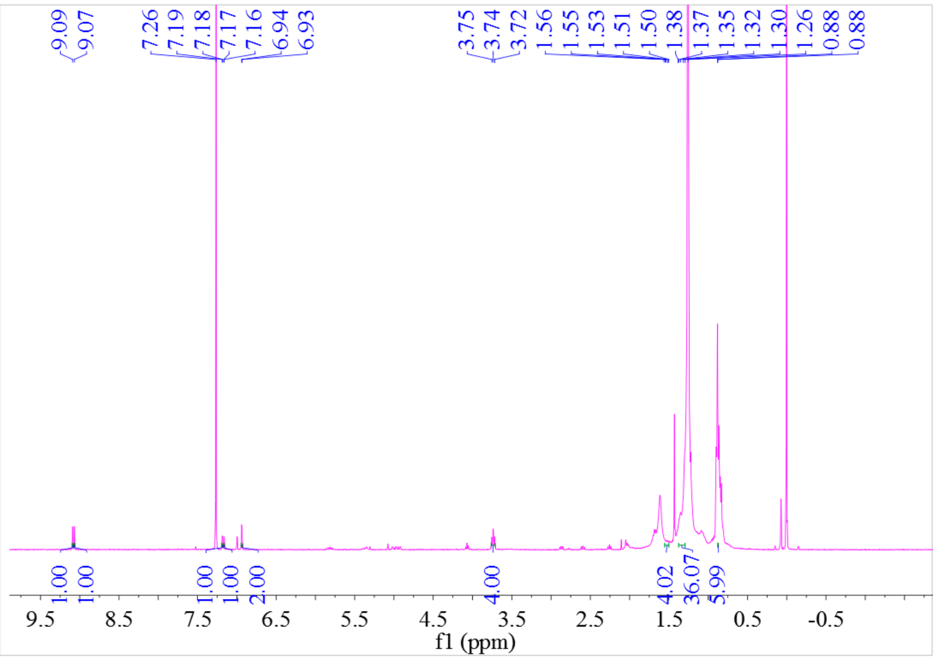

Figure S3. ^{1}H NMR spectrum of (*E*)-6,6'-dibromo-1,1'-dihexyl-[3,3'-biindolinylidene]-2,2'-dione in $CDCl_3$.

IDOH-Th: 64%. ^{1}H NMR (400 MHz, $CDCl_3$, ppm) *δ* 9.19-9.17 (d, *J* = 8.6 Hz, 4H), 7.29 (s, 4H), 6.95 (s, 4H), 3.84-3.80 (m, 4H), 2.67-2.63 (m, 4H), 1.36-1.26 (s, 12H), 0.90-0.88 (t, *J* = 6.8 Hz, 6H).

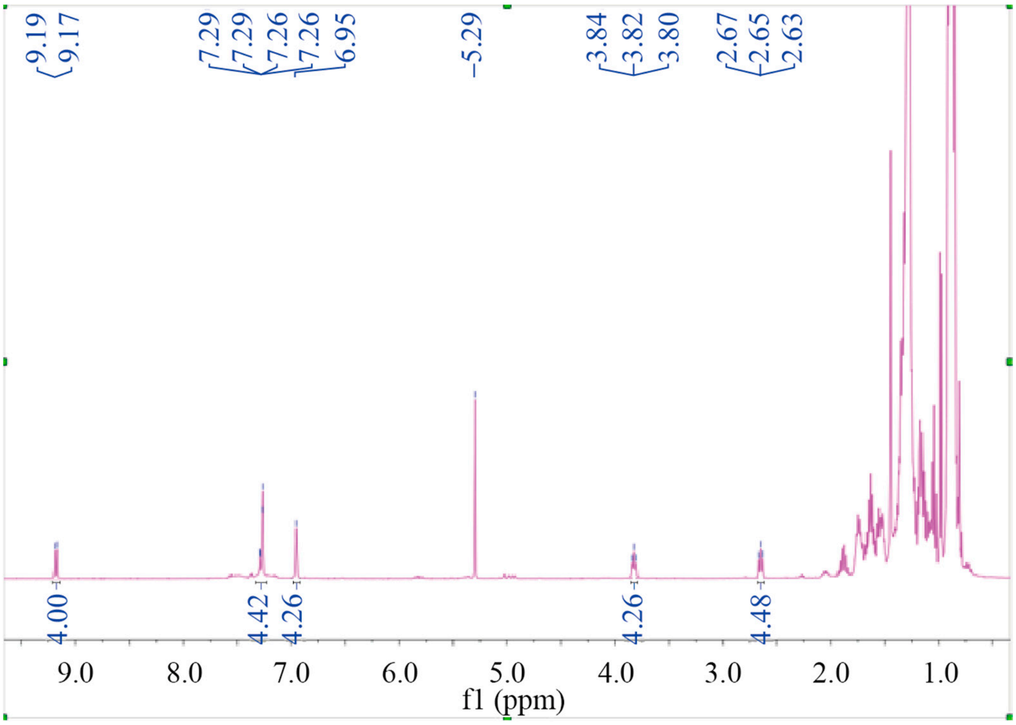

Figure S4. ^{1}H NMR spectrum of IDOH-Th in $CDCl_3$.

IDOD-Th: 62%. ^{1}H NMR (400 MHz, $CDCl_3$, ppm) δ 9.17-9.15 (d, J = 8.6 Hz, 4H), 7.28 (s, 4H), 6.96 (s, 4H), 3.84-3.81 (m, 4H), 2.66-2.62 (m, 4H), 1.74-1.65 (m, 8H), 1.87-1.85 (m, 20H), 0.92-0.88 (t, J = 6.8 Hz, 6H).

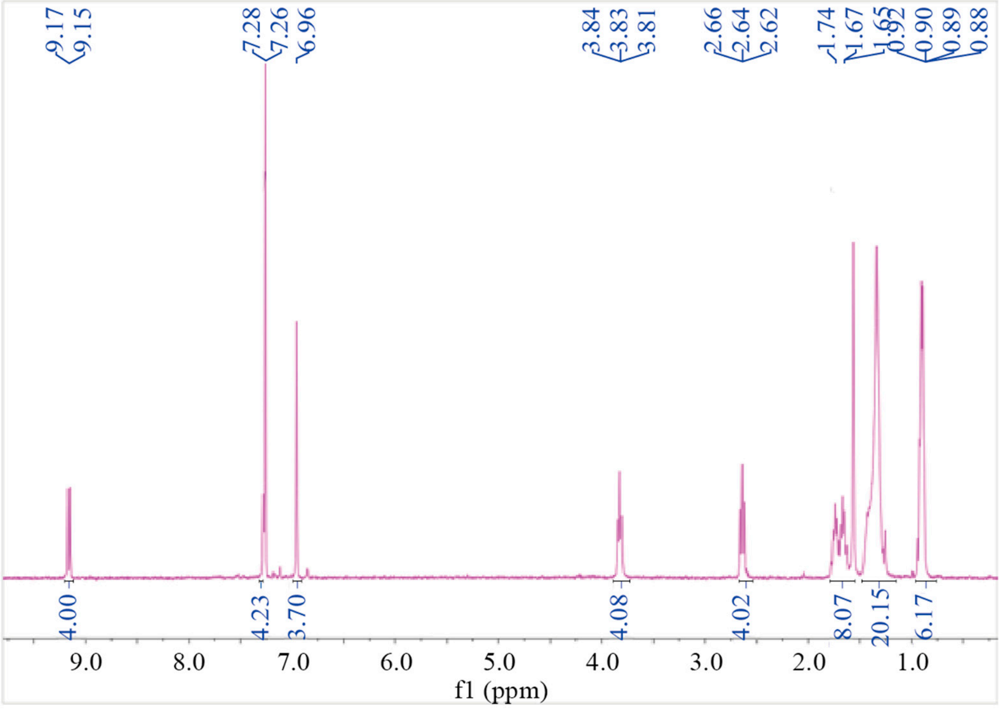

Figure S5. ^{1}H NMR spectrum of IDOD-Th in $CDCl_3$.

IDOD-Th: ^{13}C NMR (101 MHz, $CDCl_3$, ppm) *δ* 168.21, 154.30, 143.91, 137.97, 132.20, 130.46, 128.31, 126.10, 124.36, 121.15, 119.42, 104.93, 40.13, 31.93, 29.71, 29.64, 29.53, 29.34, 27.58, 27.07, 22.69, 14.10.

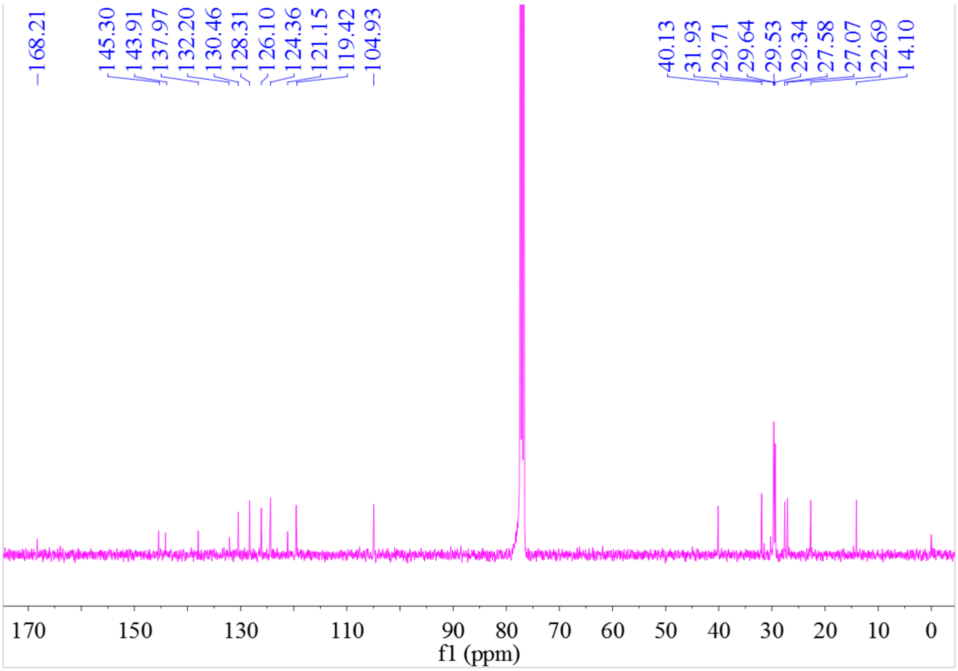

Figure S6. ^{13}C NMR spectrum of IDOD-Th in $CDCl_3$.

IDOH-3HT: 75%. ^{1}H NMR (400 MHz, $CDCl_3$, ppm) δ 9.20-9.17 (d, *J* = 8.4 Hz, 2H), 7.45-7.44 (d, *J* = 3.3 Hz, 2H), 7.37-7.36 (d, *J* = 5.0 Hz, 2H), 7.14-7.13 (d, *J* = 8.4 Hz, 2H), 6.99 (d, *J* = 4.8 Hz, 2H), 3.85 (t, *J* = 7.2 Hz, 4H), 1.76-1.72 (m, 4H), 1.35-1.27 (m, 32H), 0.88 (t, *J* = 6.5 Hz, 12H).

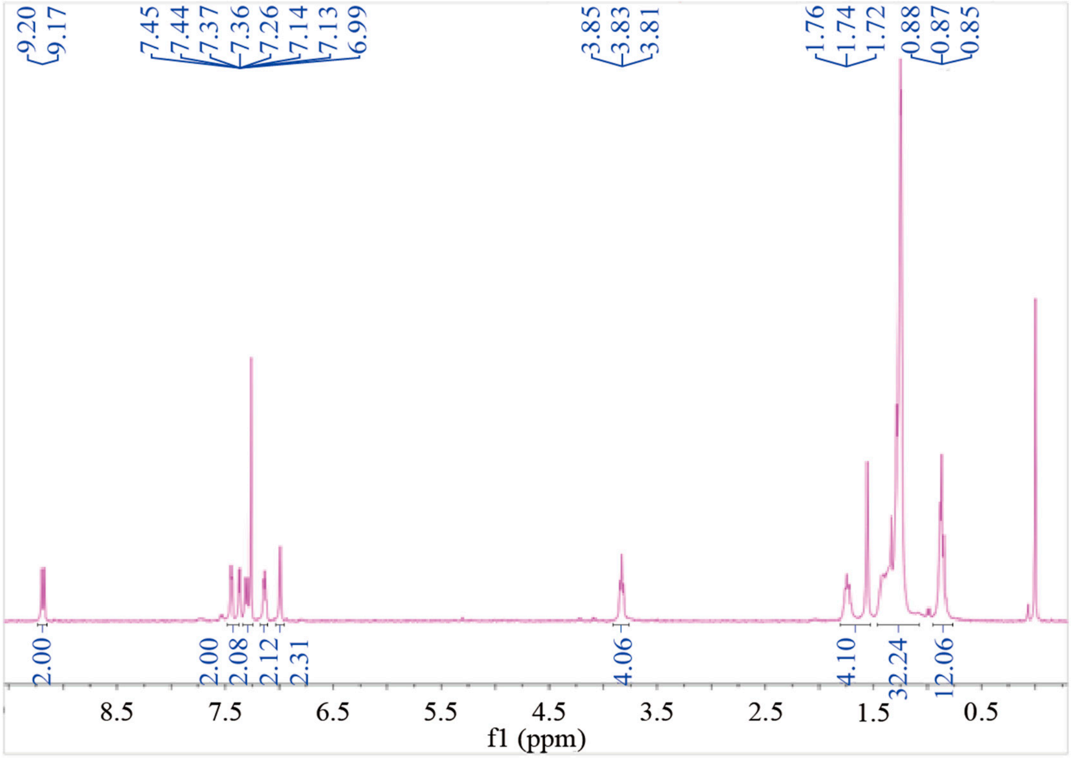

Figure S7. ^{1}H NMR spectrum of IDOH-3HT in $CDCl_3$.

IDOH-3HT: [1]C NMR (400 MHz, $CDCl_3$, ppm) δ 167.82, 144.80, 144.17, 143.14, 137.67, 131.45, 129.80, 125.19, 120.41, 118.71, 104.14, 39.55, 31.15, 30.98, 30.10, 29.92, 28.49, 27.02, 26.19, 22.09, 22.02.

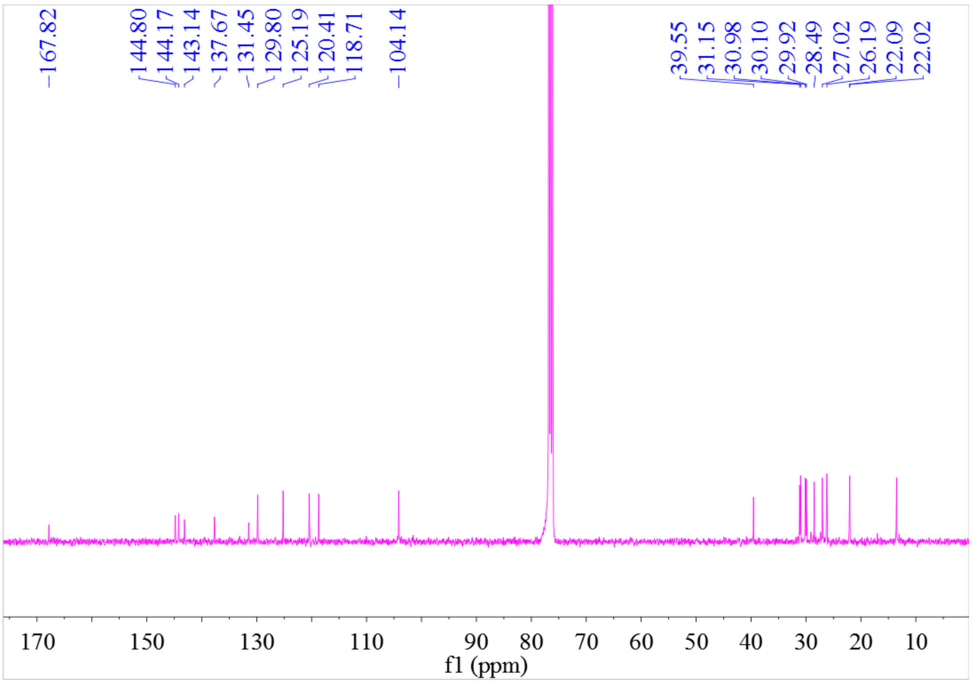

Figure S8. ^{13}C NMR spectrum of IDOH-3HT in $CDCl_3$.

IDOD-3HT: 64%. ^{1}H NMR (400 MHz, $CDCl_3$, ppm) δ 9.22-9.20 (d, J = 8.4 Hz, 2H), 7.29-7.28 (d, J = 3.3 Hz, 2H), 7.14-7.12 (d, J = 5.0 Hz, 2H), 7.02 (s, 2H), 7.01 (s, 2H), 3.82-3.79 (t, J = 7.2 Hz, 4H), 2.74-2.72 (m, 4H), 1.73-1.65 (m, 10H), 0.88-0.83 (m, 36H).

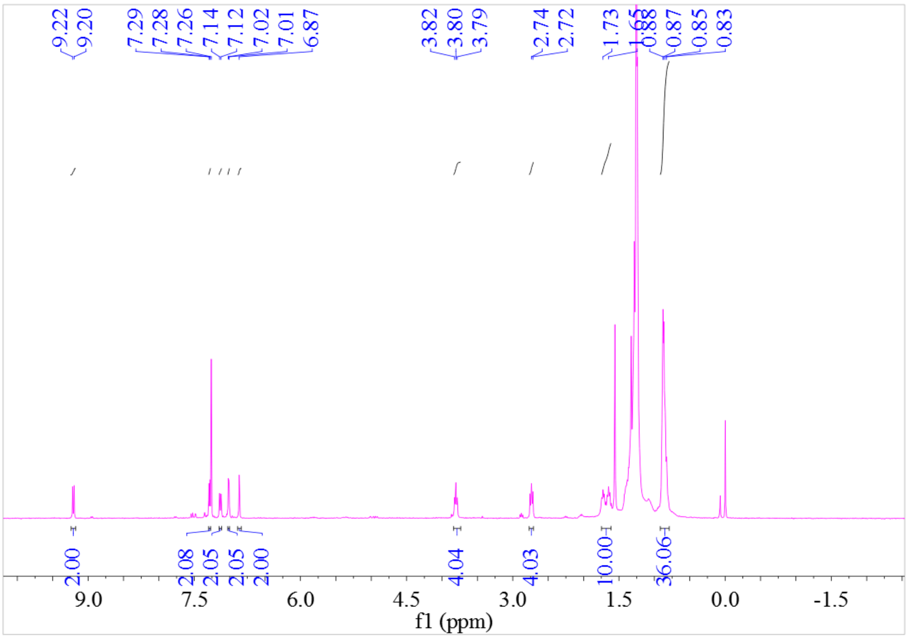

Figure S9. ^{1}H NMR spectrum of IDOD-3HT in $CDCl_3$.

(1) IDOH-Th

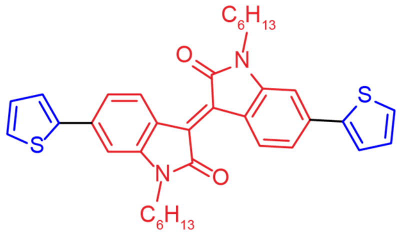

(2) IDOD-Th

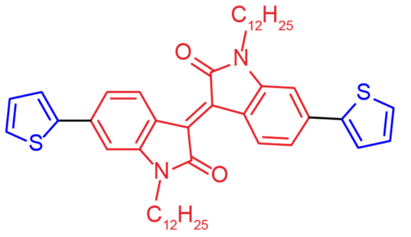

(3) IDOH-3HT

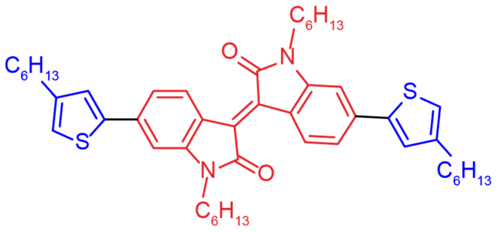

(4) IDOD-3HT

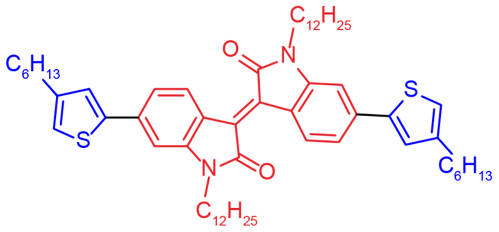

(5) IDOHE

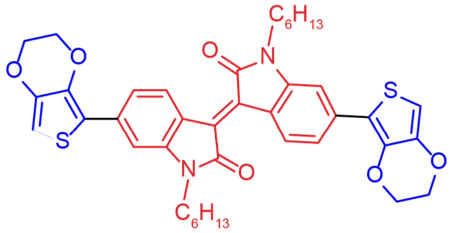

(6) IDODE

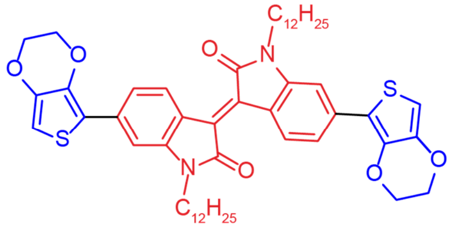

(7) EPTE

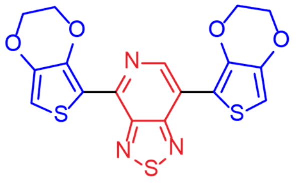

(8) EBTE

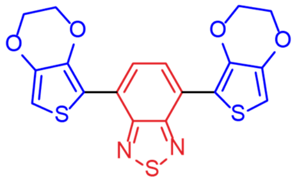

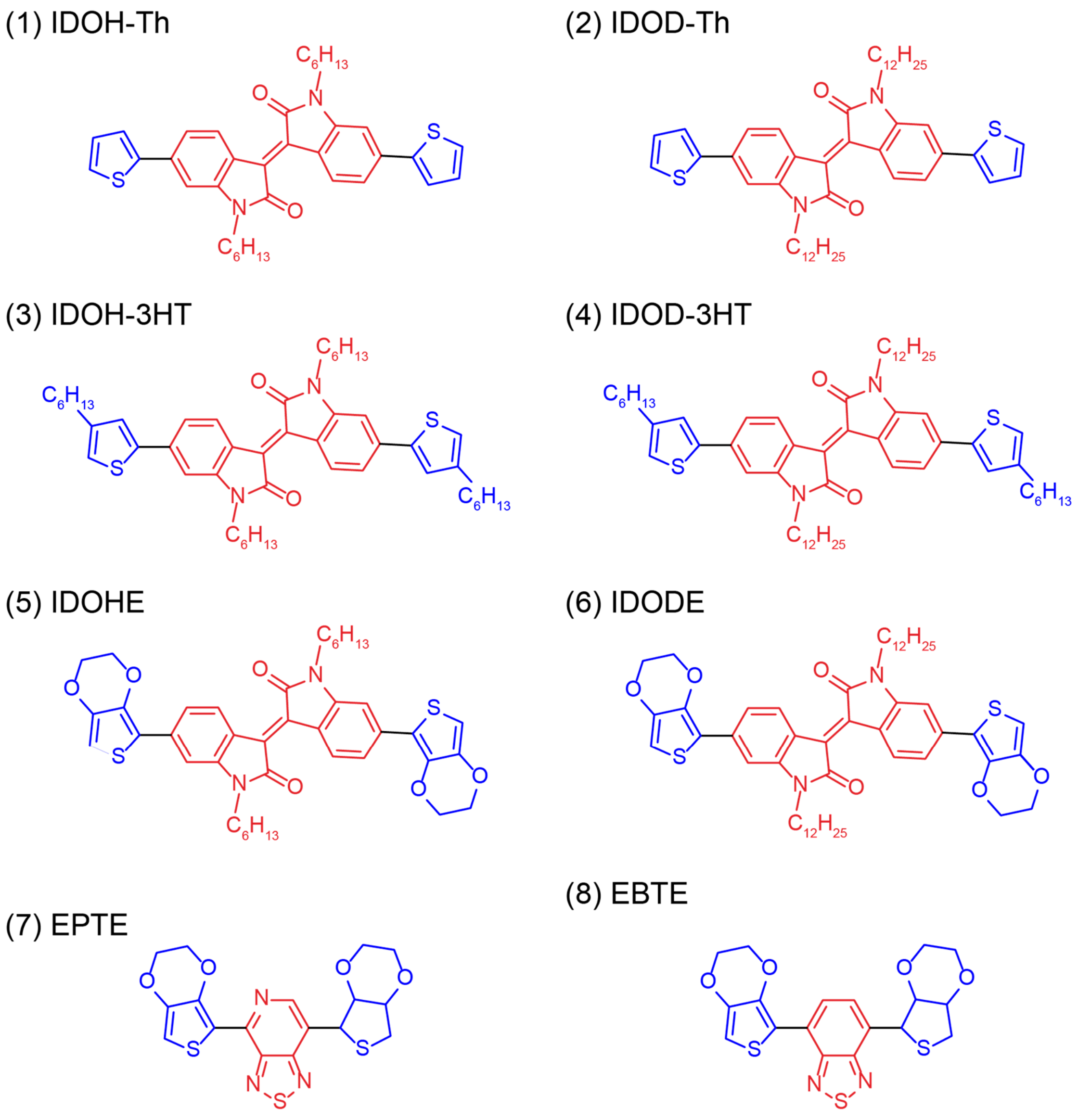

Figure S10. Chemical structures of different D-A-D type precursors.

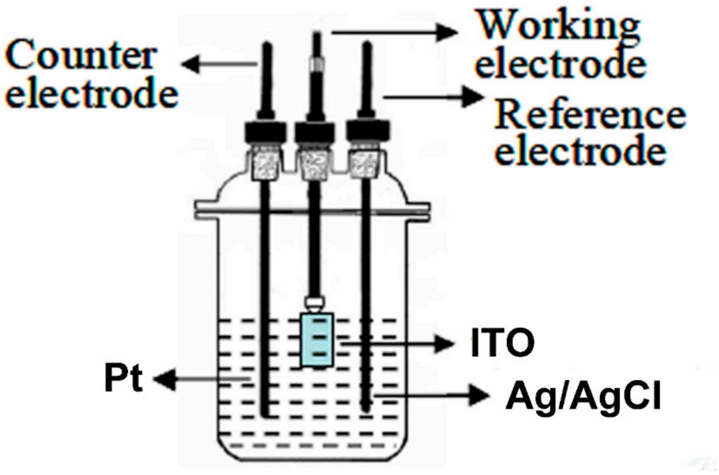

Figure S11. Schematic diagram of the equipment based on one-chamber three-electrode system.

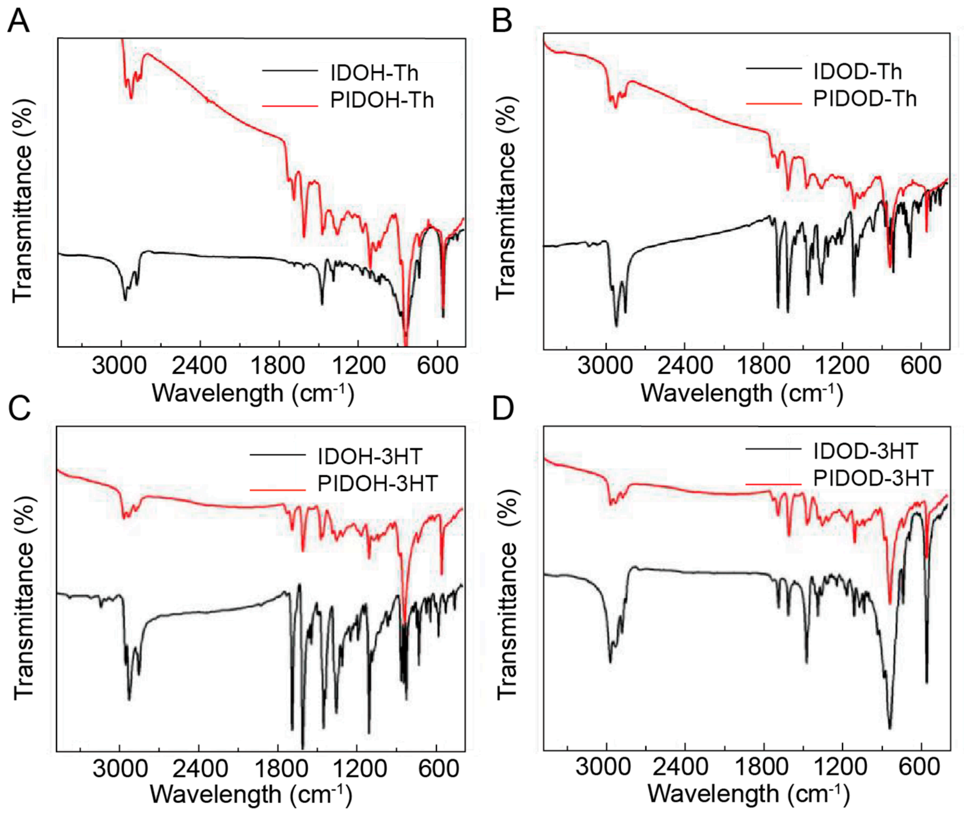

Figure S12. FT-IR spectra of precursors IDOH-Th (A), IDOD-Th (B), IDOH-3HT (C), IDOD-3HT (D), and corresponding polymers.

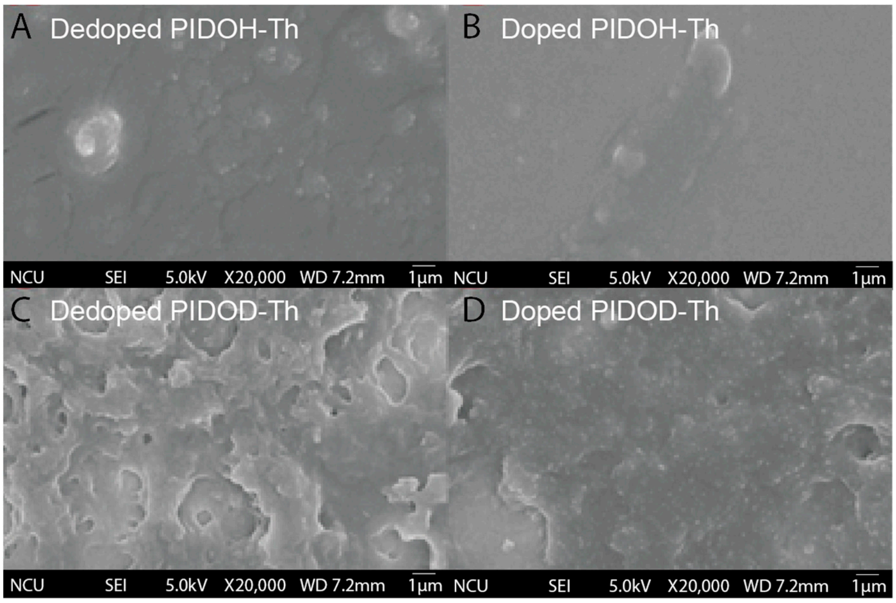

Figure S13. SEM images of PIDOH-Th and PIDOD-Th deposited electrochemically on the ITO electrode: dedoped PIDOH-Th (A) and doped PIDOH-Th (B); dedoped PIDOD-Th (C) and doped PIDOD-Th (D).

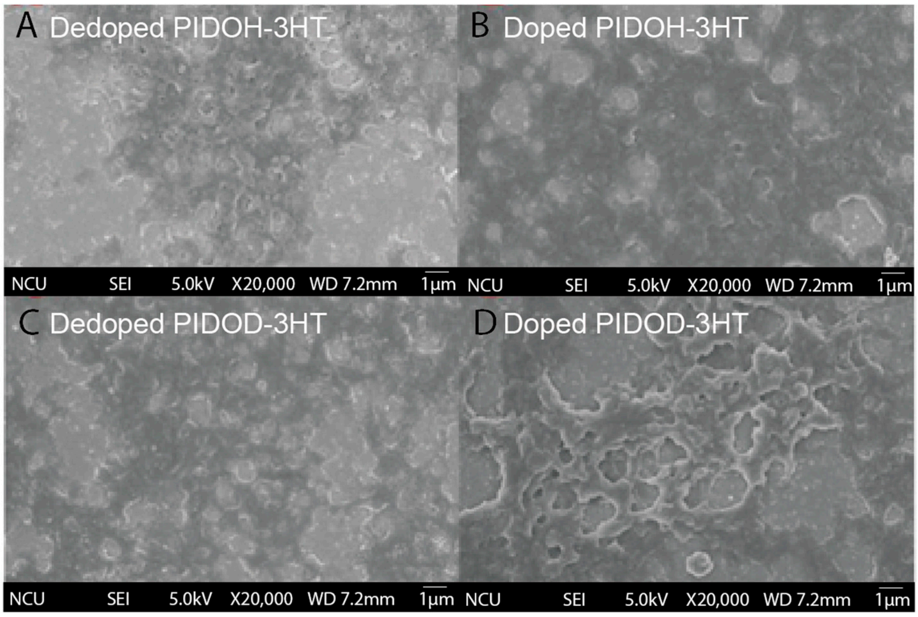

Figure S14. SEM iamges of PIDOH-3HT and PIDOD-3HT deposited electrochemically on the ITO electrode: dedoped PIDOH-3HT (A) and doped PIDOH-3HT (B); dedoped PIDOD-3HT (C) and doped PIDOD-3HT (D).

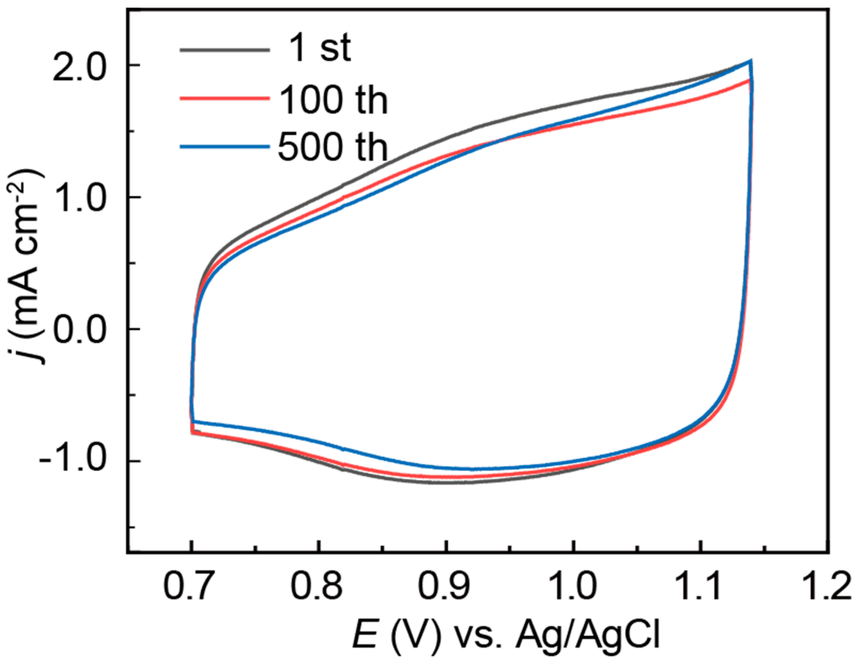

Figure S15. Long-term CVs of the deposited PIDOH-3HT film at a constant potential scan rate of 200 mV s^{-1}.

Table S1. Comparison between observed peaks of monomers and corresponding polymers

Peak (cm^{-1})		
IDOH-Th/IDOD-Th IDOH-3HT/IDOD-3HT	PIDOH-Th/PIDOD-Th PIDOH-3HT/PIDOD-3HT	Assignment
3097,3070/3090/2841/2870	--	=C-H vibration of thiophene
800/739/738/728	720/870726/821	Bending bibrations of C-H bonds on the benzene or thiophene rings
1628/1576//1605/1620	1580/1612/1595/1610	Stretching vibrations of C=C or C=O bonds or benzene rings
2903/2967/2931/2962	2838/2957/2860/2952	C-H bonds in the alkyl side chains
--	840	PF_6^-
